# Supplementary material for: Carbapenem Combinations for Infections Caused by Carbapenemase-Producing Pseudomonas aeruginosa: Experimental In Vitro and In Vivo Analysis
Source: Antibiotics (Basel). 2022 Sep 7;11(9):1212. doi: 10.3390/antibiotics11091212 (PMC9495166; doi:10.3390/antibiotics11091212)
Supplement: Supplementary file 1 [file antibiotics-11-01212-s001.zip › antibiotics-1895573-supplementary.pdf]

Supplementary Figure S1. Time-kill curves at 1xMIC of dual carbapenem combination against seven carbapenemase-producing *P. aeruginosa*.

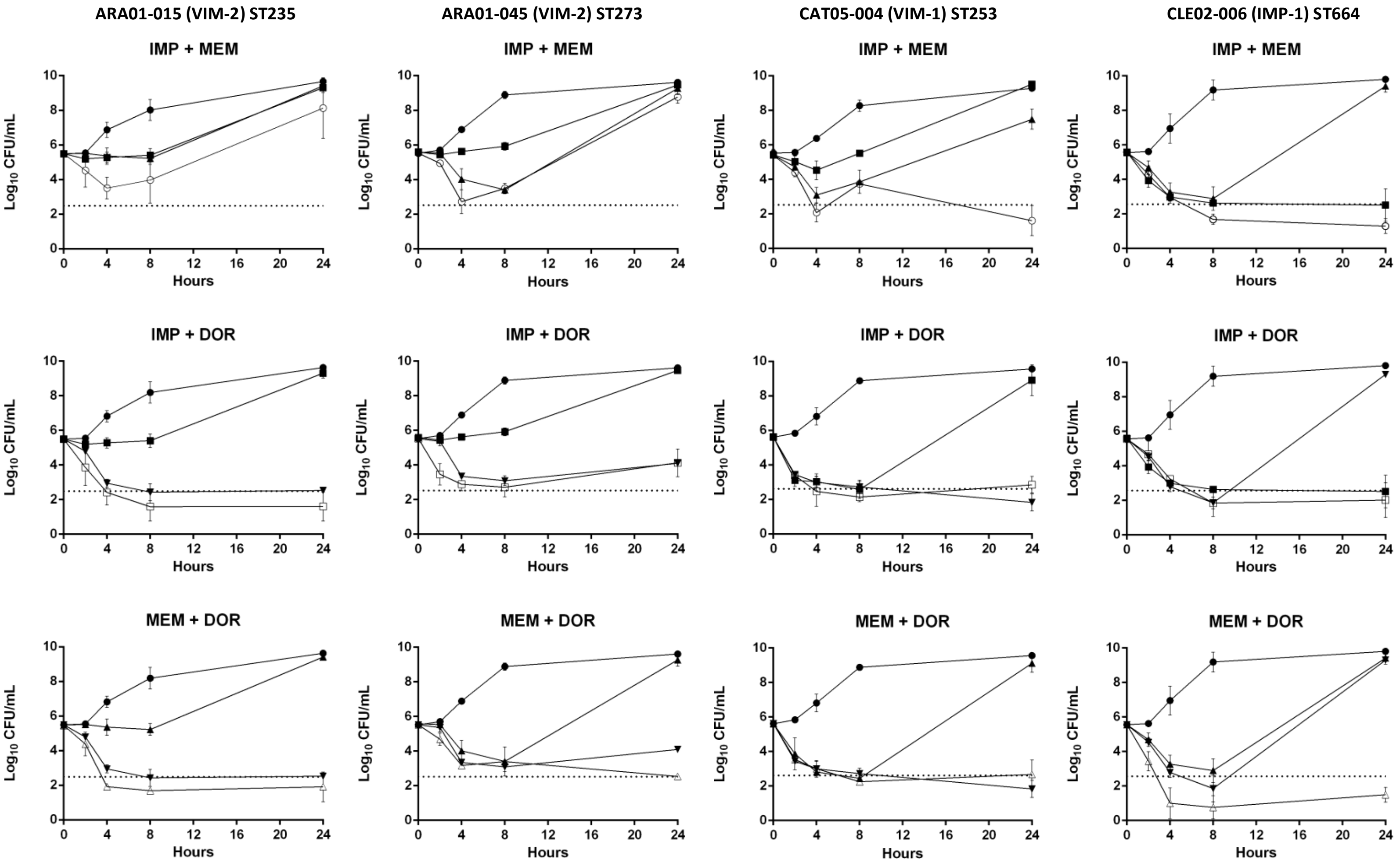

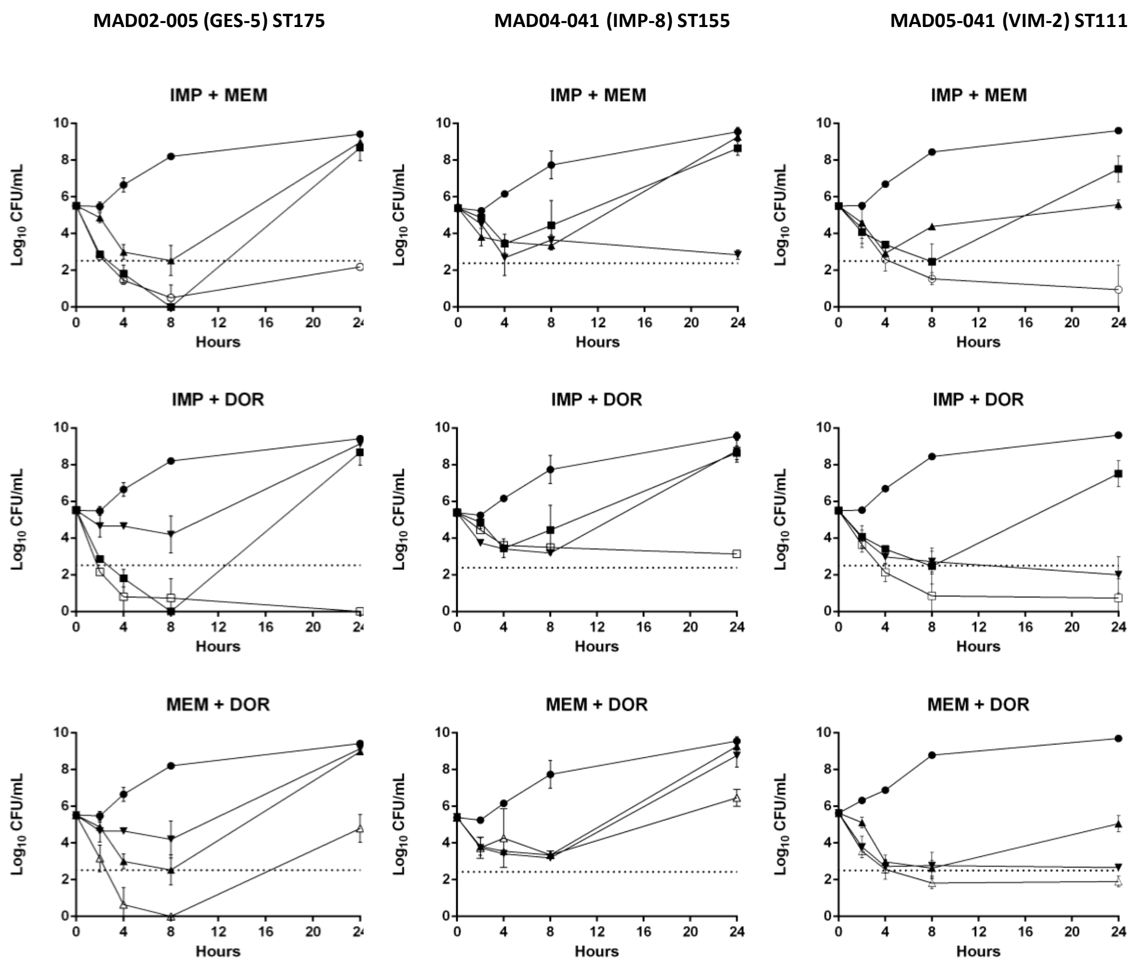

Solid circle: growth control; Square: imipenem (IMP); Triangle: meropenem (MEM); Inverted triangle: doripenem (DOR); Empty circle: IMP + MEM; Empty square: IMP + DOR; Empty triangle: MEM + DOR; Dotted line: bactericidal activity.
